# Recombinant activated factor VII (rFVIIa) as salvage treatment for intractable hemorrhage

**DOI:** 10.1186/1477-9560-2-9

**Published:** 2004-11-05

**Authors:** Anita Aggarwal, Vera Malkovska, Joseph P Catlett, Kirsten Alcorn

**Affiliations:** 1 Washington Cancer Institute, Washington Hospital Center, 110 Irving Street, NW, Washington, District of Columbia, 20010, United States of America; 2 Dept of Pathology and Laboratory Medicine, Washington Hospital Center, 110 Irving Street, NW, Washington, District of Columbia, 20010, United States of America

## Abstract

**Background:**

Recently, there has been an increased use of recombinant activated factor VII (rFVIIa) to promote hemostasis in various hemorrhagic conditions. The objective of this study was to determine the outcome of patients treated with rFVIIa who had intractable bleeding associated with cardiac surgery (CSP) or as a result of other causes (OBP).

**Methods:**

The medical records of 40 consecutive patients treated with rFVIIa were retrospectively reviewed for blood product use before and after treatment. In all patients, rFVIIa was given only after all other measures to stop bleeding had failed. The number of transfused units of red cells (R), platelets (P), fresh frozen plasma (F), and cryoprecipitate (C) were determined both before and after administration of rFVIIa, and the results compared. Mortality at 4 hours and 30 days was assessed. Patients dying within 4 hours of rFVIIa administration were not evaluable for response. Patient characteristics were also assessed as risk factors for mortality.

**Results:**

Twelve of 24 CSP survived for more than 4 hours. These 12 patients required an average of 17 units (U) of R, 18 U of P, 18 U of F and 15 U of C pre-treatment compared to an average of 6 U, 10 U, 9 U and 4 U of R, P, F and C respectively, post-treatment. These differences were statistically significant. For the OBP, 11 of 16 survived more than four hours. These 11 patients required an average of 10 U of R, 11 U of P, 14 U of F and 10 U of C pretreatment compared to an average of 1 U, 2 U, 2 U and 0 U of R, P, F, and C respectively, post-treatment. With the exception of C, there was a statistically significant decrease in blood product use following treatment with rFVIIa. Of the survivors in each group, 6 of 12 CSP and 2 of 11 OBP died between 3 and 30 days post-treatment from causes other than bleeding. Mortality at 30 days for CSP and OBP survivors was 50% and 18% respectively, whereas overall 30 day mortality was 75% for CSP and 44% for OBP.

**Conclusions:**

rFVIIa is effective in decreasing blood product use and promoting hemostasis in patients with intractable bleeding associated with cardiac surgery and a variety of other causes.

## Introduction

Recombinant factor VIIa (rFVIIa), originally developed for the treatment of acquired inhibitors associated with hemophilia [1,2], has been successfully used for bleeding due to acquired or congenital thrombocytopathy [3,4], extensive trauma and a variety of surgical procedures, including anecdotal reports of use in cardiac surgery patients [5-9]. It reverses the effect of warfarin in healthy volunteers [10], and corrects the prothrombin time in patients with hepatic failure [11].

Most critically ill patients with trauma suffer profound coagulopathy. Coagulation abnormalities in these patients have been attributed to multiple factors including disseminated intravascular coagulation (DIC), excessive fibrinolysis due to release of tissue plasminogen activator (tPA), dilutional coagulopathy from fluid replacement and massive blood product transfusion, dysfunctional platelets, and metabolic abnormalities including acidosis and hypothermia [12-18]. In addition, the use of cardiopulmonary bypass in patients undergoing cardiac surgical procedure may exacerbate the coagulopathy [19].

Although the mechanism of action of rFVIIa remains unclear, many investigators have suggested that it binds to the surface of activated platelets and directly activates Factor X, thus bypassing the early steps of the coagulation cascade. Activated Factor X (Xa) then combines with activated Factor V (Va) on the platelet surface, leading to rapid conversion of prothrombin to thrombin [20,21]. Hemostasis is promoted through high concentrations of thrombin generated near activated platelets at the site of vascular injury. Based on its mechanism of action, rFVIIa may be effective in controlling hemorrhage due to trauma, surgery and other causes.

The present report is a retrospective review of 40 consecutive patients treated with rFVIIa (NovoSeven, Novo Nordisk, Denmark) for intractable hemorrhage. Twenty-four of these patients had bleeding associated with cardiac surgery, and the remaining 16 suffered with bleeding from other causes. The need for transfusion of blood products both before and after treatment and the mortality at 4 hour and 30 day were assessed. Patient characteristics were also assessed as risk factors for mortality.

## Methods

### Patients

The study was approved by the Institutional Board Review of the Washington Hospital Center, Washington DC, USA. The medical records of 40 consecutive patients treated with rFVIIa between July 2001 and June 2003 at the Washington Hospital Center, a tertiary care teaching hospital and level 1 trauma center, were reviewed. The patients were divided into two groups: 24 patients who had undergone cardiac surgical procedures (CSP), and 16 patients who had bleeding from other causes (OBP). There were more male patients in both groups (19 males and 5 females in CSP, 10 males and 6 females in OBP). The median age in the CSP was higher at 65 years (range 26–85 years) compared to that of OBP (median age 42; range 23–82 years). The type of surgery in the CSP group included 15 coronary artery bypass grafts (CABG), 3 aortic arch repairs either elective or for dissection, 2 aorta rupture repair (one also underwent CABG), 3 valve replacements and 1 ventricular rupture. In the OBP group, 5 patients had suffered either stab or gunshot wounds, 2 had traumatic liver lacerations, 2 had undergone joint replacement, 3 had clotting factor abnormalities (acquired Factor VIII inhibitor, congenital Factor VII deficiency, acquired VWF deficiency), and one each had post partum hemorrhage, warfarin overdose, bowel resection, and trauma from a motor vehicle accident.

### Administration of rFVIIa

Commercially available rFVIIa, (Novo-Seven, Novo Nordisk, Denmark) was used in all patients. There were no predetermined criteria for administration. In most patients, treatment was given after extensive blood product support had failed to control hemorrhage, and the decision to treat was made jointly by the surgeon, hematologist and blood bank pathologist. rFVIIa was administered as a bolus dose of 90 mcg/kg. The first two patients received a second dose of 90 mcg/kg 6 hours after the first.

### Endpoints and Assessment of Risk

The total number of products given before and up to twenty-four hours after rFVIIa administration, or until death, whichever came first, was quantified. Patients who died within 4 hours of treatment were not evaluable because blood product support was suspended shortly after rFVIIa administration in these hemodynamically unstable patients deemed non-salvageable. Also, given the short half-life of rFVIIa, this time frame did not allow for an accurate determination of efficacy. The number of patients surviving at 4 hours and 30 days after the administration of rFVIIa was compared. Thirty days was chosen as an end point since this is the operative mortality cut off point, as defined by the Society of Thoracic Surgeons, for inpatient and out of hospital death. For patients surviving more than 4 hours, charts were reviewed for thromboembolic complications during hospitalization.

In an attempt to determine risk factors for poor outcome, patients were assessed for age, gender, comorbid conditions, left ventricular ejection fraction (LVEF), use of cardiopulmonary bypass (CPB), hemoglobin, platelet count, arterial blood gas results and coagulation parameters including prothrombin time (PT), activated partial thromboplastin time (aPTT), fibrinogen, and D-Dimers. Laboratory parameters were not available for some patients.

### Statistical Considerations

The Paired Student's t-Test was used to compare the number of blood product transfusions before and after treatment with rFVIIa, and, since the number of pair groups was less than 50, this result was confirmed with the Wilcoxon Matched-Pairs Signed-Ranks Test.

## Results

### Cardiac Surgery Patients (CSP)

#### Outcome

Twelve of the 24 patients survived for more than 4 hours after the administration of rFVIIa, and the results of their pre- and post-treatment blood product use are listed in Table 1. From the start of surgery until the administration of rFVIIa, patients received on average 17 U of R (range 5–39), 18 U of P (range 6–37), 18 U of F (range 6–33), and 15 U of C (range 0–50). Post rFVIIa treatment, an average of 6 U of R (range 0–28), 10 U of P (range 0–19), 9 U of F (range 0–28) and 4 U of C (0–20) were transfused. The difference between the pre- and post-transfusion requirement was significantly lower for all blood products: R (p = 0.007), P (p = 0.038), F (p = 0.009) and C (p = 0.03). Eight of the 12 patients showed a rapid hemostatic response to rFVIIa, as measured by a decrease in R transfusion to an average of 2 U (range 0–6). The other 4 patients (Patient # 24, 27, 32 and 35) continued to require blood product support beyond 24 hours after rFVIIa administration.

**Table 1:** Summary of rFVIIa treated cardiac surgery patients (CSP) who survived >4 hours

**P#**	**Age sex**	**Procedure**	**Co-morbidities**		**Response to rFVIIa**								**Outcome**
				
			**CPB**	**EF%**	**Pre rFVIIa**				**Post rFVIIa**				
						
					**R**	**P**	**F**	**C**	**R**	**P**	**F**	**C**	
1	75M	CABG	-	>45	18	18	8	10	0	4	0	0	Survived
13	36M	Aortic arch repair	+	>45	28	18	16	10	6	19	20	10	Died
14	64M	Aortic dissection	-	>45	39	37	32	50	2	6	4	0	Survived
17	63M	AVR/MVR repair	-	<25	7	18	16	15	1	12	6	0	Survived
18	70M	CABG	+	>25	6	20	20	20	0	0	0	0	Survived
20	72M	CABG	-	>25	21	12	10	0	0	0	0	0	Survived
24*	79M	CABG	+	>25	10	12	12	10	5	4	4	0	Died
27*	64M	CABG, IVAD	+	<25	30	30	33	20	28	16	28	20	Died
30	85M	CABG, ruptured aorta	+	>45	13	12	6	10	3	6	8	0	Died
32*	83M	CABG	+	>25	5	18	18	10	6	18	20	0	Died
33	78F	Ruptured aorta	+	<25	14	18	20	20	3	12	6	0	Died
35*	54M	Aortic arch repair	+	>45	9	6	18	10	15	24	12	20	Survived
**Average**					**17**	**18**	**18**	**15**	**6**	**10**	**9**	**4**	
**P value**									**.009**	**.038**	**.009**	**.03**	

P: Patient

R: Red Blood Cell; P: Platelet; F: Fresh Frozen Plasma; C: Cryoprecipitate

M: Male; F: Female

CPB: Cardiopulmonary Bypass

EF: Left Ventricular Ejection Fraction

CABG: Coronary Artery Bypass Graft

AVR: Aortic Valve Replacement

MVR: Mitral Valve Replacement

IVAD: Intraventricular Assisted Device

* Patient continued to require blood transfusion past 24 hours post rFVIIa treatment.

Six of the 12 patients who survived more than four hours were eventually discharged to home (Table 1). The remaining 6 patients (Patient # 13, 24, 27, 30, 32, and 33) died between 3 and 30 days after rFVIIa treatment from a variety of causes including multi-organ failure and infections. Amongst survivors beyond 4 hours, mortality at 30 days was 50% and overall mortality for the entire group was 75%.

Of the 12 patients who died from multiorgan failure and hemodynamic instability within 4 hours of receiving rFVIIa, 11 died within 2 hours and 1 died within 4 hours (8 during surgery and 4 in the recovery room). Due to the limited time frame, it was not possible to determine if bleeding in these patients diminished after rFVIIa treatment. In the urgent setting in which these patients were treated, determining the amount and timing of blood loss and blood transfusion was difficult. Moreover, for patients deemed non-salvageable, blood product transfusion was suspended before response to rFVIIa could be determined.

#### Risk factors and outcome in CSP

Overall, 17 of 24 CSP were over the age of 60. Twenty underwent CPB, and 15 had a LVEF below 25%. Fourteen underwent mediastinal re-exploration for bleeding. Twenty-three patients had a significant coagulopathy defined as thrombocytopenia below 100,000/mm^3^, prolonged PT and PTT or both (data not given due to wide variations in the results). Seven of the 24 patients did not have a documented arterial blood gas (ABG) around the time of rFVIIa administration; however, among the other 17 patients, pH's of 7.0 in 1, 7.2 in 5, and 7. 4 in 11 patients were noted. With regards to rFVIIa administration, there was no correlation between pH, PT, PTT, thrombocytopenia, anemia and outcome.

Of the 12 patients who died within 4 hours, all underwent CPB for over 30 minutes (time on CPB for 8 patients was more than 2.5 hours). All 12 had an LVEF of less than 25% and 7 had mediastinal re-exploration for bleeding. In contrast, only 3 of the 12 surviving patients had EF <25%, 8 had surgery with CPB and 7 underwent mediastinal re-exploration. The median age and coagulation parameters were not different between the two groups.

### Other Bleeding Patients (OBP)

#### Outcome

Eleven of the 16 patients with bleeding due to other causes survived for more than 4 hours after rFVIIa administration, and the results of their blood product use before and after rFVIIa are summarized in Table 2. From the beginning of surgery or time of trauma until the administration of rFVIIa, patients received an average of 10 U of R (range 2–28), 11 U of P (range 0–36), 14 U F (range 0–34) and 10 U of C (range 0–70). Post administration of rFVIIa, an average of 1 U of R (range 0–6), 2 U of P (range 0–6), 2 U of F (0–10) and 0 U of C were transfused over 24 hours. The difference between the pre- and post-rFVIIa treatment blood product requirement was significantly lower for R (p = 0.004), P (p = 0.017), and F (p = 0.017), but not for C (p = 0.122). Nine of the 11 showed a rapid response to rFVIIa, as measured by a decrease in R transfusion to an average of <0.5 U (range 0–3). The other 2 patients (Patient # 2 and 15) continued to require blood product support beyond 24 hours post rFVIIa treatment.

**Table 2:** Summary of rFVIIa treated other bleeding patients (OBP) who survived >4 hours

**P#**	**Age** **Sex**	**Procedure**	**Co-morbidities**	**Response to rFVIIa**								Outcome
					
				**Pre rFVIIa**				**Post rFVIIa**				
					
				**R**	**P**	**F**	**C**	**R**	**P**	**F**	**C**	
2*	48F	Colectomy	Colon cancer	4	6	24	70	2	0	2	0	Died
15*	26F	Cystectomy, nephrectomy, pelvic fracture, termination of Pregnancy	MVA, 20 weeks gestation	18	12	8	0	6	6	10	0	Survived
16	53M	Left femur fixation	Multiple myeloma	12	12	6	0	0	0	0	0	Survived
19	82M	Shoulder replacement	HTN, CAD, Renal failure	7	10	20	10	2	6	4	0	Survived
21	54F	Liver laceration	Trauma	23	17	34	20	3	6	10	0	Survived
22	24M	Neck soft tissue trauma	FVII deficiency	2	0	4	0	0	0	0	0	Survived
23	64F	Liver laceration	Dialysis	28	36	33	0	0	0	0	0	Died
28	30F	Postpartum hemorrhage	None	12	18	6	10	0	0	0	0	Survived
29	64M	Gastrointestinal bleed	FVIII inhibitor	2	0	0	0	0	0	0	0	Survived
38	60M	Thyroidectomy	VWF deficiency	4	12	12	4	0	0	0	0	Survived
40	40F	Splenic subcapsular bleed	Amyloidosis, coumadin overdose	4	0	8	0	0	0	0	0	Survived
**Average**				**10**	**11**	**14**	**10**	**1**	**2**	**2**	**0**	
**P value**								**.004**	**.017**	**.017**	**.122**	

P: Patient

R: Red Blood Cell;

P: Platelet; F: Fresh Frozen Plasma; C: Cryoprecipitate

M: Male; F: Female

EF: Left Ventricular Ejection Fraction

VWF: von Willebrand Factor

* Patient continued to require blood transfusion past 24 hours post rFVIIa treatment.

Nine of the 11 surviving patients were discharged to home and 2 (Patient # 2 and 23) died between 3 and 30 days from a combination of infection, cardiac arrest and multiorgan failure. Amongst survivors beyond 4 hours, mortality at 30 days was 18% and overall mortality for the entire group was 44%.

All five of the 16 patients who died within four hours of rFVIIa treatment had massive injuries either from gunshot or stab wounds. Upon arrival to the emergency room, these patients were hemodynamically unstable, with hemoglobin values ranging between 3–6 g/dL. rFVIIa was given as a last resort, but these patients were not evaluable since transfusions were suspended shortly after infusion of rFVIIa.

#### Risk factors and outcome in OBP

The median age of this group was 42 years. None underwent CPB, and two of the 7 patients tested had an EF <25%. Only 1 patient had a second exploratory laparotomy for bleeding. The patient suffering with a warfarin overdose had a splenic subcapsular bleed complicating systemic amyloidosis.

At the time of rFVIIa administration, a wide variation in laboratory parameters was observed in these patients (data not shown). There was no difference in coagulation parameters between patients who died and those who survived. Eight of the 16 patients did not have an ABG documented. In the 5 patients who died within four hours, the pH was lower (6.9–7.0) than for those who survived (7.3–7.4).

### Thromboembolism after rFVIIa

Only one of the 23 patients in both the CSP and OBP groups who survived more than 4 hours had a documented thromboembolic complication after receiving rFVIIa. Deep vein thrombosis (DVT) of the left subclavian vein was confirmed by a Doppler ultrasound 2 days after rFVIIa administration. The thrombus was related to a central line placed before rFVIIa treatment. No other clinical or laboratory evidence of thromboembolism was documented in the medical records of the other patients throughout their hospital stays. None of the other 17 patients in both the groups who died within 4 hours were evaluable for thromboembolic complications.

## Discussion

This retrospective case series suggests that rFVIIa is effective in promoting hemostasis even when given as a last life-saving measure in poor prognosis patients with massive, transfusion-refractory hemorrhage. A statistically significant decrease in blood product transfusion was evident in 12 CSP and 11 OBP who survived more than 4 hours after rFVIIa infusion. Six of 12 CSP and 2 of 11 OBP died later between 3 and 30 days after rFVIIa infusion, but these deaths were due to causes other than hemorrhage.

Despite the obvious limitations of a retrospective analysis, this is the largest series of such patients reported in the literature to date. Al Douri et al [8], described 5 patients who underwent open-heart surgery for valvular disease and experienced uncontrollable intraoperative or postoperative bleeding unresponsive to a blood product support protocol. The study was prospective, had well-defined criteria for "excessive bleeding" and excluded patients with a history of ischemic heart disease, stroke or venous thromboembolism. Hemostasis was achieved in all patients following a single dose of 30 ug/kg of rFVIIa, and there was no mortality from bleeding, although 1 patient died 3 days later of an unrelated cause. Overall, the 4-hour and 30 day mortality in the present series was high compared to that of Al Douri et al [8], but it is difficult to compare these two very different patient populations.

Factors predictive of inferior outcome after cardiac surgery include age over 60, low LVEF, significant co-morbidity, the use of CPB and re-exploration after initial surgery [22-24]. The use of CPB increases the rate of fibrinolysis and induces an inflammatory response [19,25]. Moreover, prolonged time on CPB, defined as 2.5 hours or more, is associated with increased bleeding and need for re-exploration. Two-6% of patients undergoing CABG require re-exploration for bleeding and is associated with a mortality rate of up to 22% [19,26,27]. In this series, the CSP group had multiple poor prognostic factors. The median age was 65 years and in addition, all 12 of the CSP who died within 4 hours and 3 of 6 CSP who died later had preoperative LVEF of less than 25%. All of the 12 CSP who died within 4 hours in this series, required CPB and 8 of these patients were on CPB for more than 2.5 hours. More than half of our CSP (14 of 24) required re-exploration, which undoubtedly influenced the mortality rate.

Both, the CSP and OBP groups had a significant decrease in blood product use after rFVIIa treatment but the short and long-term survival appeared to be worse in CSP probably due to multiple poor prognostic factors as discussed above. Poor outcome has also been independently associated with peri-operative transfusion of more than 7 units of R [28]. The CSP and OBP who survived received an average of 18 and 11 units of R in the peri-operative period, respectively. The R requirement of patients who died within 4 hours in both groups was probably higher but could not be reliably estimated. All 5 of the patients in the OBP group who died within 4 hours suffered from major blood vessel injury as a result of stabbing or gun shot wounds. These patients were unstable upon arrival to the emergency room with hemoglobins in the range of 3–6 g/dL.

Excessive hemorrhage requiring massive transfusion can lead to hypothermia, DIC, excessive fibrinolysis, dilutional coagulopathy, and metabolic acidosis, which may further exacerbate bleeding and morbidity. [14-17] A reduction in the pH to 7.0 nearly abolishes rFVIIa activity as reported by Meng et al [18]. In this present series, there was no obvious correlation between the coagulopathy and mortality in both the CSP and OBP groups. Five of the OBP who died within 4 hours had a pH of 7 or less. However, acidosis did not appear to play an important role in CSP.

The optimal dose and timing of administration of rFVIIa for these patients is unknown. The standard dose (90 mcg/kg) was first used in hemophiliacs and was based on the *in vitro *dose-dependent reduction in aPTT in plasma from these patients [1,29]. The 90 mcg/kg dose of rFVIIa corresponds to concentration of 1.25 mcg/ml FVIIa in plasma. Martinowitz et al [7], used a median dose of 120 mcg/kg (range 120–210 mcg/kg) to achieve hemostasis in 7 cases of trauma. In 2 patients, 120 mcg/kg was insufficient, and 2 additional doses of 60 mcg/kg were required to achieve hemostasis. Kenet et al. [30], have shown better efficacy with a 300 mcg/kg bolus dose followed by continuous infusion. The standard therapeutic dose of 90 mcg/kg of rFVIIa was used in this series of patients although a lower dose of 30 mcg/kg dose has also been shown to be effective [8]. However, in considering the data from the aforementioned studies, it would appear that higher initial doses, or additional doses, might improve outcome. It is also tempting to speculate that earlier treatment with rFVIIa may have prevented rapid clinical deterioration and complications associated with massive blood product transfusion in this series of patients. This question would best be addressed in controlled studies using standard dosing protocols and well defined criteria for intractable hemorrhage. Although rFVIIa is expensive, it would appear to be cost effective when compared with the combined cost of large amount of blood products.

Previous clinical experience with rFVIIa supports a good safety profile in patients with hemophilia and trauma. Less than 1% of patients receiving rFVIIa had thrombosis and thrombosis related complications [21,31]. In our series, only one patient had a deep vein thrombosis of the left arm associated with an indwelling line and not considered to be due to rFVIIa. There were no other documented cases of thrombosis or microemboli.

## Conclusions

This retrospective study suggests that rFVIIa can play a beneficial role as an adjunctive hemostatic agent in patients after cardiac surgery or extensive trauma who experience bleeding that cannot be controlled by conventional therapies. Prospective studies are necessary to determine optimal patient selection, and dose and timing of rFVIIa administration.
